# Reply to Kagias and Stampanoni: High-sensitivity hard X-ray directional differential phase imaging

**DOI:** 10.1073/pnas.2116067118

**Published:** 2021-11-15

**Authors:** Hongchang Wang, Kawal Sawhney

**Affiliations:** ^a^Diamond Light Source Ltd., Harwell Science and Innovation Campus, Didcot, OX11 0DE, United Kingdom

Kagias and Stampanoni (1) claim that there are a few passages that are inaccurate in our recent publication (2). We dispute this claim. We agree that the directional derivates can be calculated by having access to two orthogonal components; in fact, this is already described and included in our publication (2).

The multiple directional differential phase images (DDPI) can be directly measured with the method proposed (2), and the amplitude $A_{1}\;$ differential phase and the phase term $\phi_{A}$ can be derived with fast Fourier transform (FFT) analysis. The DDPI at any angle *θ* can be expressed as follows (equation 4 in ref. 2):

[1] $$\alpha^{\theta }=-A_{1}\cos\left(\text{θ}+\phi_{A}\right)=-A_{1}\cos\;\theta \;\text{cos}\;\phi_{A}+A_{1}\sin\theta \;\text{sin}\;\phi_{A}\;.$$

According to equation 5 in ref. 2, the horizontal and vertical DPI can be calculated.

[2] $$\{\begin{array}{l} \alpha^{0}=-A_{1}\;\text{cos}(\phi_{A})\\ \alpha^{\pi /2}=A_{1}\;\text{sin}(\phi_{A})\; \end{array}.$$

Combining the two equations gives us the following simplified equation:

[3] $$\alpha^{\theta }=\alpha^{0}\cos\left(\theta \right)+\alpha^{\pi /2}\;\text{sin}(\theta ),$$

although Eq. **3** is mathematically the same as cited by Kagias and Stampanoni (1), who suggest using only horizontal and vertical DDPI (*n* = 2) to calculate the value at any angle *θ*. In contrast, we can measure multiple DDPI (*n* > 2), and the averaged horizontal and vertical DDPI can be derived using equation 2 in our work (2). There are two main advantages: 1) The artifacts for DDPI can be minimized from multiple images, and 2) a higher signal-to-noise ratio can be achieved.

**Fig. 1.**
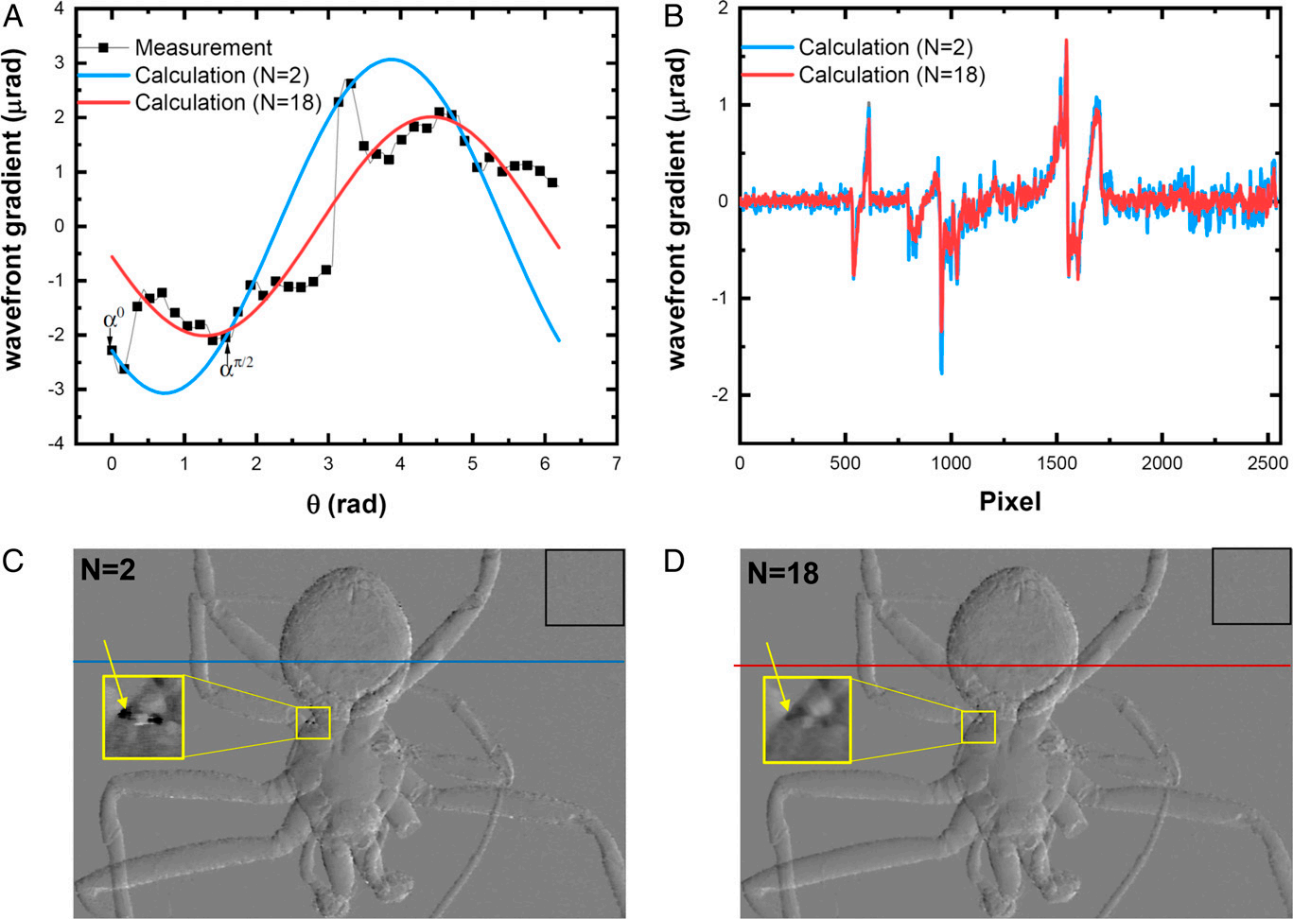
(*A*) Measured wavefront gradient in one pixel (inside of yellow circle in *C*) and the calculated values with Eq. 3 with number of DDPI *n* = 2 (proposed in ref. 1) and *n* = 18 (described in ref. 2). (*B*) The line profile of the calculated DDPI *n* = 2 and *n* = 18, and (*C* and *D*) the calculated DDPI at angle θ=π/9  with *n* = 2 and *n* = 18.

Fig. 1*A* shows the measured differential phase (black) along multiple directions (*n* = 18 over π) at a single pixel (pointed to by the yellow arrow in Fig. 1 *C* and *D*), and the calculated differential phase with only two angles (blue) and 18 angles (red). As expected, the artifacts present at the two angles will be transferred to the other DDPI. In contrast, such artifacts are significantly reduced by applying FFT analysis over multiple images (*n* = 18). The calculated DDPI at angle θ=π/9  with *n* = 2 and *n* = 18 is shown in Fig. 1 *C* and *D*. The artifacts in the yellow rectangle in Fig. 1*C* still exist, while they are hardly visible for the one with the multiple images (*n* = 18). The line profile from the two methods is compared in Fig.1*B*, and the data are less noisy for the multiple images (*n* = 18) compared to with only two images; the SDs in the square black box are 0.14 μrad (*n* = 2) and 0.07 μrad (*n* = 18), respectively.

Kagias and Stampanoni (1) seem to have wrongly concluded that we imply that the already published methodology for omnidirectional scattering imaging is limited and lacking practical implementations. On the contrary, we have acknowledged their important work on practical implementation of directional dark-field imaging by citing two of their publications (references 13 and 16 in ref. 2). As the practical implementations are very few so far (3), we expect that our work (2) that includes both differential dark-field imaging and differential phase contrast imaging will significantly increase further practical imaging applications. And that is what we wanted to convey by *“*the presented technique could potentially open up numerous practical imaging applications in both biomedical research and materials science.” In hindsight, perhaps prefixing or replacing “numerous” with “further” in the above sentence would have been more appropriate.
